# Crystal structures of the dioxane hemisolvates of *N*-(7-bromo­methyl-1,8-naphthyridin-2-yl)acetamide and bis­[*N*-(7-di­bromo­methyl-1,8-naphthyridin-2-yl)acetamide]

**DOI:** 10.1107/S2056989017012208

**Published:** 2017-09-05

**Authors:** Robert Rosin, Wilhelm Seichter, Monika Mazik

**Affiliations:** a Institut für Organische Chemie, Technische Universität Bergakademie Freiberg, Leipziger Strasse 29, D-09596 Freiberg/Sachsen, Germany

**Keywords:** crystal structure, 1,8-naphthyridine, dioxane solvates, hydrogen bonding, C—Br⋯O=C inter­actions: C—Br⋯π inter­actions, halogen bonds, π–π stacking

## Abstract

The syntheses and crystal structures of *N*-(7-bromo­methyl-1,8-naphthyridin-2-yl)acetamide dioxane hemisolvate, C_11_H_10_BrN_3_O·0.5C_4_H_8_O_2_, (I), and bis­[*N*-(7-di­bromo­methyl-1,8-naphthyridin-2-yl)acetamide] dioxane hemisolvate, 2C_11_H_9_Br_2_N_3_O·0.5C_4_H_8_O_2_, (II), are described. The mol­ecules adopt a conformation with the N—H hydrogen pointing towards the lone electron pair of the adjacent naphthyridine N atom. The crystals of (I) are stabilized by a three-dimensional supra­molecular network comprising N—H⋯N, C—H⋯N and C—H⋯O hydrogen bonds, as well as C—Br⋯π halogen bonds. The crystals of compound (II) are stabilized by a three-dimensional supra­molecular network comprising N—H⋯N, C—H⋯N and C—H⋯O hydrogen bonds, as well as C—H⋯π contacts and C—Br⋯π halogen bonds. The structure of the substituent attached in the 7-position of the naphthyridine skeleton has a fundamental influence on the pattern of inter­molecular noncovalent bonding. While the Br atom of (I) participates in weak C—Br⋯O_guest_ and C—Br⋯π contacts, the Br atoms of compound (II) are involved in host–host inter­actions *via* C—Br⋯O=C, C—Br⋯N and C—Br⋯π bonding.

## Abstract 2

The syntheses and crystal structures of the title dioxane hemisolvates of *N*-(7-bromo­methyl-1,8-naphthyridin-2-yl)acetamide and bis­[*N*-(7-di­bromo­methyl-1,8-naphthyridin-2-yl)acetamide] are described.

## Chemical context   

In recent decades, 1,8-naphthyridines have attracted increasing inter­est because of their biological and medicinal activities (Ferrarini *et al.*, 1998 ▸; Roma *et al.*, 2010 ▸; Badaweh *et al.*, 2001 ▸; Litvinov, 2004 ▸), as ligands in the synthesis of metal complexes (Tang *et al.*, 2015 ▸; Matveeva *et al.*, 2013 ▸; Kolotuchin & Zimmerman, 1998 ▸) and as building blocks for various supra­molecular systems (Kolotuchin & Zimmerman, 1998 ▸; Park *et al.*, 2005 ▸; Liang *et al.*, 2012 ▸). Compound (I) represents a useful precursor for the synthesis of artificial receptor mol­ecules, for example, for carbohydrate receptors bearing naphthyridine units (Mazik & Cavga, 2007 ▸; Mazik & Sicking, 2001 ▸; Cuntze *et al.*, 1995 ▸).

## Structural commentary   

**Figure 1:**
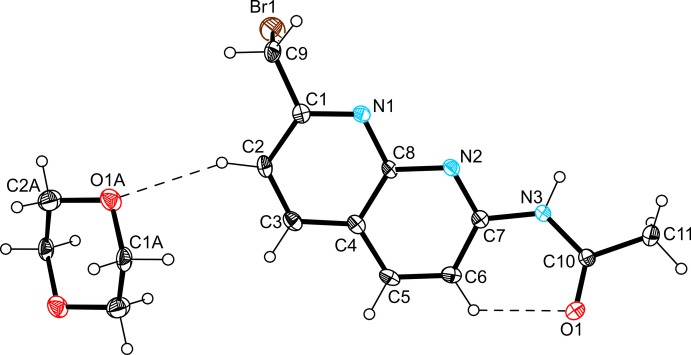
A view of the mol­ecular structure of compound (I), showing the atom labelling. Displacement ellipsoids are drawn at the 50% probability level. Dashed lines represent halogen bonds (Table 1 ▸).

The mol­ecular structures of the title compounds, (I) and (II), are illustrated in Figs. 1 ▸ and 2 ▸, respectively. The asymmetric unit of compound (I) consists of one mol­ecule of the naphthyridine derivative and one half of a 1,4-dioxane solvent mol­ecule, with the whole mol­ecule being generated by inversion symmetry. The naphthyridine ring of the host mol­ecule is essentially planar [maximum deviations from the mean plane being 0.034 (3) Å for N1 and −0.034 (3) Å for C6]. The plane defined by the acetamido group is inclined at an angle of 18.9 (2)° with respect to the mean plane of the 1,8-naphthyridine moiety. The torsion angle along the atomic sequence N2—C1—C9—Br1 is 83.6 (4)°. The dioxane mol­ecule is connected to the host mol­ecule *via* C—H⋯O hydrogen bonding (Table 1 ▸ and Fig. 1 ▸).
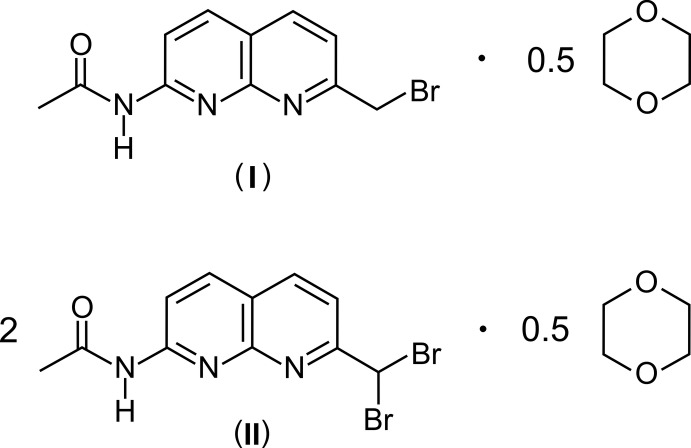


**Figure 2:**
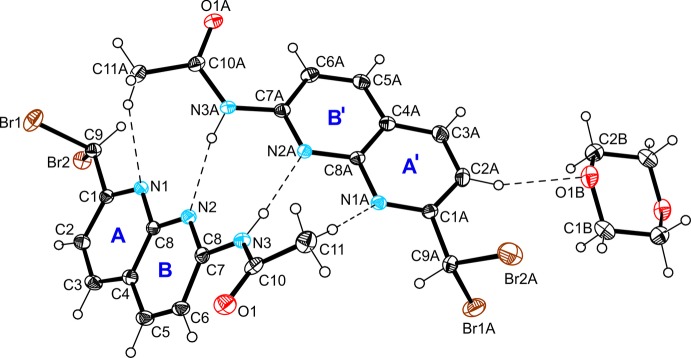
A view of the two independent mol­ecules of compound (II), showing the atom labelling and ring specification. Displacement ellipsoids are drawn at the 50% probability level. For the sake of clarity, the minor-disordered component of the dioxane mol­ecule has been omitted. Dashed lines represent hydrogen bonds (Table 2 ▸).

The asymmetric unit of the inclusion compound (II) contains two crystallographically independent, but conformationally similar mol­ecules of the 1,8-naphthyridine derivative and one half mol­ecule of a positionally disordered 1,4-dioxane, the whole mol­ecule of the latter is generated by inversion symmetry and is disordered over two positions [occupancy ratio = 0.890 (5):0110 (5)]. The structural features of the host mol­ecule in (II) resemble those found in the reported structure of *N*-(7-di­bromo­methyl-5-methyl-1,8-naphthyridin-2-yl)acetamide (Gou *et al.*, 2013 ▸). The dihedral angles between the mean planes of the naphthyridine moiety and the acetyl­amido group are 27.6 (1) and 20.4 (1)°, respectively. The di­bromo­methyl group is oriented in such a way that the two Br atoms are tilted away from the plane of the respective naphthyridine moiety. The dioxane mol­ecule is connected to the host mol­ecule *via* C—H⋯O hydrogen bonding (Table 2 ▸ and Fig. 2 ▸).

## Supra­molecular features   

**Figure 3:**
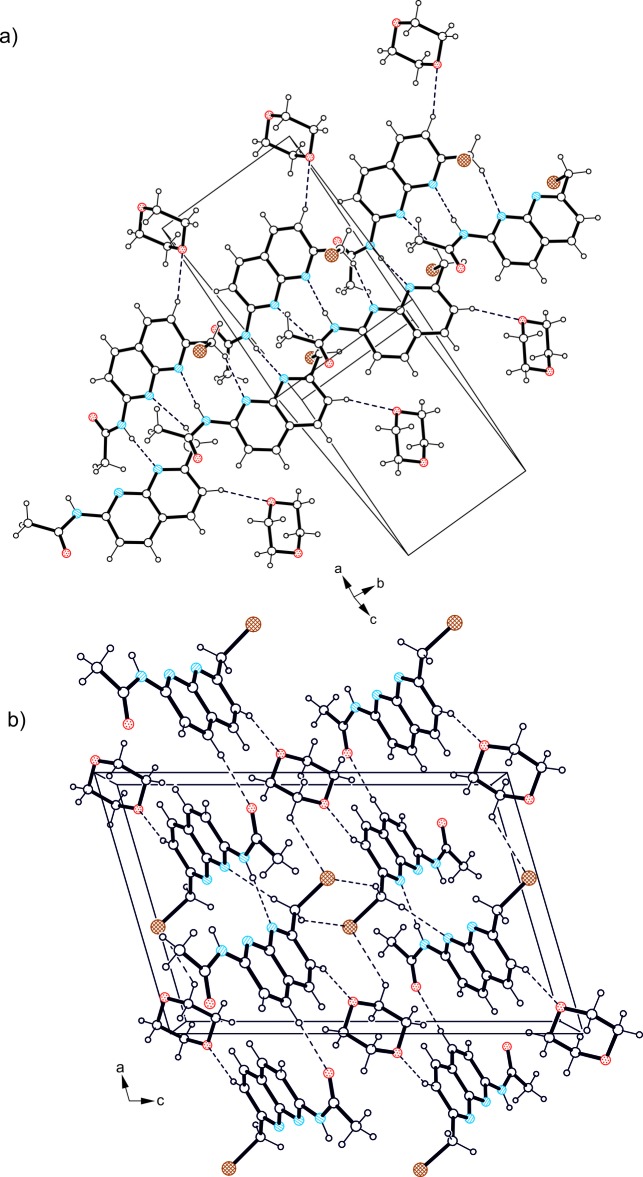
A view of the crystal packing of compound (I) (*a*) normal to the 101 plane and (*b*) along the *b* axis. Dashed lines represent hydrogen bonds.

In the crystal of compound (I), 1:1 host–guest units related by the 2_1_ screw axis are linked *via* hydrogen bonding to form infinite supra­molecular strands (Fig. 3 ▸ and Table 1 ▸). In this mol­ecular arrangement, the amino H atom and atom N2 participate in inter­molecular N—H⋯N hydrogen bonding, whereas atom N1 is involved in the formation of a weaker C—H⋯N inter­action with one of the methyl­ene H atoms of a symmetry-related mol­ecule acting as a donor. These hydrogen bonds create a loop with graph-set motif 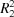
(8). An inter­strand inter­action is accomplished by C_arene_—H⋯O and C—H⋯Br hydrogen bonds, as well as weak C—Br⋯π [C—Br⋯C_naph_ = 3.527 (2) Å and 170.1 (1)°] contacts, thus creating a three-dimensional supra­molecular architecture.

**Figure 4:**
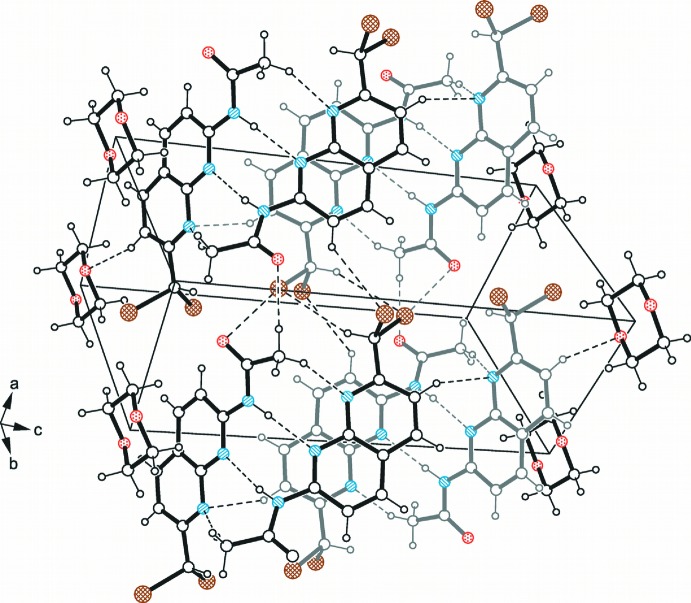
A view of the crystal packing of compound (II). For the sake of clarity, the minor component of the disordered dioxane mol­ecule has been omitted. Dashed lines represent hydrogen and halogen bonds.

According to the observed stoichiometric ratio of the crystal components in (II), the host mol­ecules contribute in a different way in noncovalent inter­molecular bonding. The crystal structure is constructed of 2:1 host–guest complexes (Fig. 2 ▸ and Table 2 ▸), in which the independent host mol­ecules form a strongly distorted dimer held together by two N—H⋯N hydrogen bonds and two weak C_meth­yl_—H⋯N contacts. One of the arene H atoms of this dimeric unit acts as a donor for C—H⋯O hydrogen bonding to the guest mol­ecule. As is shown in Fig. 4 ▸ and Table 2 ▸, the Br atoms of only one host mol­ecule participate in inter­molecular inter­actions. Atom Br1 is involved in the formation of a weak C—H⋯Br contact. Moreover, the Br1⋯*Cg*(B) distance of 3.317 (2) Å and the well-defined bonding geometry [C9—Br1⋯*Cg*(B) = 173.0 (1)°] indicate the presence of an inter­molecular Br⋯π halogen bond (Mazik *et al.*, 2010*a* ▸,*b* ▸; Koch *et al.*, 2017 ▸; Legon, 1999 ▸; Megrangolo & Resnati, 2008 ▸). The distance of 3.213 (2) Å between atom Br2 and amide atom O1*A* of an adjacent mol­ecule [symmetry code: (A) *x* + 1, *y* − 1, *z*], which is considerably less than the sum of the van der Waals radii of the respective atoms (3.37 Å), suggests the existence of an attractive Br⋯O halogen bond (Politzer *et al.*, 2007 ▸; Koch *et al.*, 2014 ▸, 2015 ▸). One of the host mol­ecules participates in offset π–π stacking [*Cg*⋯*Cg* = 3.709 (2) Å; symmetry code: −*x*, −*y*, −*z* + 1]. The combination of these inter­actions results in the formation of a three-dimensional supra­molecular network.

## Database survey   

The search of the Cambridge Structural Database (Groom *et al.*, 2016 ▸; Version 5.38, last update February 2017) for com­pounds representing 7-substituted 2-(*N*-acyl­amino)-1,8-naphthyridines including solvates/hydrates resulted in 14 hits. Of particular inter­est are the unsolvated crystal structures of *N*-(7-methyl-1,8-naphthyridin-2-yl)acetamide (Goswami *et al.*, 2007 ▸), and *N*-(7-chloro-1,8-naphthyridin-2-yl)acetamide and *N*-(7-chloro-1,8-naphthyridin-2-yl)butanoyl­amide (Ghosh *et al.*, 2010 ▸). These two compounds (space group *P*2_1_/*c*) reveal mol­ecular assemblies similar to that observed for compound (I), *viz*. forming infinite chains of hydrogen-bonded mol­ecules, whereas the enhanced steric demand of the butanoyl group of the latter compound favours dimer formation.

## Synthesis and crystallization   

*N*-(7-Methyl-1,8-naphthyridin-2-yl)acetamide (9.67 g, 48.1 mmol), *N*-bromosuccinimide (9.07 g, 55.6 mmol) and 2,2′-azobisisobutyronitile (AIBN; 0.10 g, 0.6 mmol), dissolved in 300 ml of dry chloro­form, were refluxed for 8 h with vigorous stirring in the presence of light from a 500 W lamp. The succinimide precipitate was filtered off and the organic filtrate washed several times with water. After drying of the filtrate over anhydrous Na_2_SO_4_ and removing the solvent, the crude product [a mixture containing *N*-(7-bromo­methyl-1,8-naphthyridin-2-yl)acetamide and *N*-(7-di­bromo­methyl-1,8-naphthyridin-2-yl)acetamide] was purified by column chro­matography (SiO_2_, eluent: ethyl acetate).

*N*-(7-Bromo­methyl-1,8-naphthyridin-2-yl)acetamide: white solid (2.56 g). ^1^H NMR (500 MHz, CDCl_3_): δ 2.29 (*s*, 3H, CH_3_), 4.70 (*s*, 2H, CH_2_), 7.59 (*d*, *J* = 8.3 Hz, 1H, CH_Ar_), 8.16 (*d*, *J* = 8.3 Hz, 1H, CH_Ar_), 8.19 (*d*, *J* = 8.8 Hz, 1H, Ar), 8.54 (*d*, *J* = 8.8 Hz, 1H, CH_Ar_), 8.93 (*s*, 1H, NH). ^13^C NMR (125 MHz, CDCl_3_): δ 25.1, 33.8, 115.6, 119.7, 120.9, 137.7, 139.3, 153.8, 154.0, 160.8, 169.7.

*N*-(7-Di­bromo­methyl-1,8-naphthyridin-2-yl)acetamide: white solid (3.20 g). ^1^H NMR (500 MHz, CDCl_3_): δ 2.31 (*s*, 3H, CH_3_), 6.87 (*s*, 1H, CH), 7.98 (*d*, *J* = 8.4 Hz, 1H, CH_Ar_), 8.21 (*d*, *J* = 8.8 Hz, 1H, CH_Ar_), 8.26 (*d*, *J* = 8.4 Hz, 1H, CH_Ar_), 8.59 (*d*, *J* = 8.8 Hz, 1H, CH_Ar_), 8.97 (*s*, NH). ^13^C NMR (125 MHz, CDCl_3_): δ 25.0, 41.6, 116.2, 119.4, 120.3, 138.6, 139.1, 152.4, 154.6, 161.8, 169.6.

Crystals of (I) and (II) suitable for X-ray analysis were obtained by slow evaporation of the solvent (1,4-dioxane) from solutions of the respective compounds.

## Refinement   

Crystal data, data collection and structure refinement details are summarized in Table 3 ▸. In both compounds, the N—H H atoms were located from difference Fourier maps and refined freely. C-bound H atoms were placed geometrically and allowed to ride on their attached C atoms, with C—H distances of 0.95–1.00 Å and *U*_iso_(H) = 1.5*U*_eq_(C-meth­yl), or 1.2*U*_eq_(C) for other H atoms.

## Supplementary Material

Crystal structure: contains datablock(s) I, II, Global. DOI: 10.1107/S2056989017012208/su5386sup1.cif

Structure factors: contains datablock(s) I. DOI: 10.1107/S2056989017012208/su5386Isup4.hkl

Structure factors: contains datablock(s) II. DOI: 10.1107/S2056989017012208/su5386IIsup5.hkl

CCDC references: 1570289, 1570288

Additional supporting information:  crystallographic information; 3D view; checkCIF report

## Figures and Tables

**Table 1:** Hydrogen- and halogen-bond geometry (Å, °) for (I) *Cg*1 and *Cg*2 are the centroids of rings N1/C1–C4/C8, and N2/C4–C8, respectively.

*D*—H⋯*A*	*D*—H	H⋯*A*	*D*⋯*A*	*D*—H⋯*A*
C6—H6⋯O1	0.95	2.29	2.836 (6)	116
N3—H3*A*⋯N2^i^	0.88 (1)	2.18 (1)	3.053 (5)	173 (5)
C1*A*—H1*A*1⋯O1^ii^	0.99	2.55	3.462 (6)	153
C2—H2⋯O1*A* ^iii^	0.95	2.54	3.438 (5)	157
C5—H5⋯O1^ii^	0.95	2.48	3.376 (6)	157
C9—H9*A*⋯N1^iv^	0.99	2.47	3.418 (6)	161
C9—Br1⋯*Cg*1^v^	1.94 (1)	3.70 (1)	5.563 (5)	161 (1)
C9—Br1⋯*Cg*2^vi^	1.94 (1)	3.70 (1)	5.436 (5)	148 (1)

Symmetry codes: (i) 
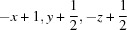
; (ii) 
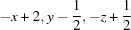
; (iii) 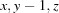; (iv) 
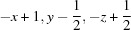
; (v) 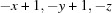; (vi) 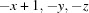.

**Table 2:** Hydrogen- and halogen-bond geometry (Å, °) for (II) *Cg*1, *Cg*2 and *Cg*4 are the centroids of rings N1/C1–C4/C8, N2/C4–C8 and N2*A*/C4*A*–C8*A*, respectively.

*D*—H⋯*A*	*D*—H	H⋯*A*	*D*⋯*A*	*D*—H⋯*A*
C6—H6⋯O1	0.95	2.37	2.886 (4)	113
C6*A*—H6*A*⋯O1*A*	0.95	2.32	2.871 (5)	116
N3—H3⋯N2*A* ^i^	0.89 (3)	2.10 (3)	2.985 (4)	171 (4)
N3*A*—H3*A*⋯N2^i^	0.89 (3)	2.09 (3)	2.950 (4)	163 (3)
C2—H2⋯N1*A*	0.95	2.56	3.397 (4)	147
C2*A*—H2*A*⋯O1*B* ^ii^	0.95	2.42	3.344 (5)	163
C11—H11*B*⋯N1*A* ^i^	0.98	2.44	3.411 (5)	169
C11*A*—H11*D*⋯O1^iii^	0.98	2.46	3.437 (5)	179
C11*A*—H11*E*⋯N1^i^	0.98	2.54	3.459 (4)	156
C3—H3*AA*⋯*Cg*4	0.95	2.82	3.548 (3)	134
C2*BA*—H2*B*3⋯*Cg*4^iv^	0.99	2.96	3.82 (9)	145
C9—Br1⋯*Cg*1^ii^	1.94 (1)	3.62 (1)	5.270 (4)	141 (1)
C9—Br1⋯*Cg*2^ii^	1.94 (1)	3.32 (1)	5.247 (4)	173 (1)

Symmetry codes: (i) 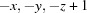; (ii) 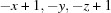; (iii) 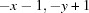, 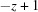; (iv) 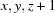.

**Table 3:** Experimental details

	(I)	(II)
Crystal data		
Chemical formula	C_11_H_10_BrN_3_O·0.5C_4_H_8_O_2_	2C_11_H_9_Br_2_N_3_O·0.5C_4_H_8_O_2_
*M*_r_	324.19	762.11
Crystal system, space group	Monoclinic, *P*2_1_/*c*	Triclinic, *P* 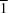
Temperature (K)	100	100
*a*, *b*, *c* (Å)	10.8863 (10), 7.6256 (7), 16.5300 (15)	9.4065 (5), 9.5271 (5), 16.6464 (10)
α, β, γ (°)	90, 106.310 (4), 90	88.777 (3), 81.057 (2), 64.928 (2)
*V* (Å^3^)	1317.0 (2)	1333.15 (13)
*Z*	4	2
Radiation type	Mo *K*α	Mo *K*α
μ (mm^−1^)	3.12	6.08
Crystal size (mm)	0.34 × 0.06 × 0.06	0.40 × 0.18 × 0.09

Data collection		
Diffractometer	Bruker APEXII CCD area detector	Bruker APEXII CCD area detector
Absorption correction	Multi-scan (*SADABS*; Bruker, 2014 ▸)	Multi-scan (*SADABS*; Bruker, 2014 ▸)
*T*_min_, *T*_max_	0.417, 0.835	0.195, 0.611
No. of measured, independent and observed [*I* > 2σ(*I*)] reflections	9345, 2493, 1965	32569, 5659, 5247
*R*_int_	0.043	0.025
(sin θ/λ)_max_ (Å^−1^)	0.610	0.636

Refinement		
*R*[*F* ^2^ > 2σ(*F* ^2^)], *wR*(*F* ^2^), *S*	0.051, 0.144, 1.03	0.029, 0.074, 1.12
No. of reflections	2493	5659
No. of parameters	177	354
No. of restraints	1	6
H-atom treatment	H atoms treated by a mixture of independent and constrained refinement	H atoms treated by a mixture of independent and constrained refinement
Δρ_max_, Δρ_min_ (e Å^−3^)	0.93, −0.85	0.92, −0.80

Computer programs: *APEX2* (Bruker, 2014 ▸), *SAINT* (Bruker, 2014 ▸), *SHELXS97* (Sheldrick, 2008 ▸), *SHELXL2013* (Sheldrick, 2015 ▸), *ORTEP-3 for Windows* (Farrugia, 2012 ▸) and *SHELXTL* (Sheldrick, 2008 ▸).

## supplementary crystallographic information

### N-(7-Bromomethyl-1,8-naphthyridin-2-yl)acetamide dioxane hemisolvate (I) Crystal data

**Table:**

C_11_H_10_BrN_3_O·0.5C_4_H_8_O_2_	*F*(000) = 656
*M**_r_* = 324.19	*D*_x_ = 1.635 Mg m^−^^3^
Monoclinic, *P*2_1_/*c*	Mo *K*α radiation, λ = 0.71073 Å
*a* = 10.8863 (10) Å	Cell parameters from 1901 reflections
*b* = 7.6256 (7) Å	θ = 3.3–25.6°
*c* = 16.5300 (15) Å	µ = 3.12 mm^−^^1^
β = 106.310 (4)°	*T* = 100 K
*V* = 1317.0 (2) Å^3^	Needle, colourless
*Z* = 4	0.34 × 0.06 × 0.06 mm

### N-(7-Bromomethyl-1,8-naphthyridin-2-yl)acetamide dioxane hemisolvate (I) Data collection

**Table:**

CCD area detector diffractometer	1965 reflections with *I* > 2σ(*I*)
phi and ω scans	*R*_int_ = 0.043
Absorption correction: multi-scan (SADABS; Bruker, 2014)	θ_max_ = 25.7°, θ_min_ = 2.0°
*T*_min_ = 0.417, *T*_max_ = 0.835	*h* = −13→9
9345 measured reflections	*k* = −8→9
2493 independent reflections	*l* = −14→20

### N-(7-Bromomethyl-1,8-naphthyridin-2-yl)acetamide dioxane hemisolvate (I) Refinement

**Table:**

Refinement on *F*^2^	Primary atom site location: structure-invariant direct methods
Least-squares matrix: full	Secondary atom site location: difference Fourier map
*R*[*F*^2^ > 2σ(*F*^2^)] = 0.051	Hydrogen site location: mixed
*wR*(*F*^2^) = 0.144	H atoms treated by a mixture of independent and constrained refinement
*S* = 1.03	*w* = 1/[σ^2^(*F*_o_^2^) + (0.074*P*)^2^ + 4.7912*P*] where *P* = (*F*_o_^2^ + 2*F*_c_^2^)/3
2493 reflections	(Δ/σ)_max_ < 0.001
177 parameters	Δρ_max_ = 0.93 e Å^−^^3^
1 restraint	Δρ_min_ = −0.85 e Å^−^^3^

### N-(7-Bromomethyl-1,8-naphthyridin-2-yl)acetamide dioxane hemisolvate (I) Special details

**Table:**

Geometry. All esds (except the esd in the dihedral angle between two l.s. planes) are estimated using the full covariance matrix. The cell esds are taken into account individually in the estimation of esds in distances, angles and torsion angles; correlations between esds in cell parameters are only used when they are defined by crystal symmetry. An approximate (isotropic) treatment of cell esds is used for estimating esds involving l.s. planes.

### N-(7-Bromomethyl-1,8-naphthyridin-2-yl)acetamide dioxane hemisolvate (I) Fractional atomic coordinates and isotropic or equivalent isotropic displacement parameters (Å^2^)

**Table:**

	*x*	*y*	*z*	*U*_iso_*/*U*_eq_	
Br1	0.40285 (5)	0.22955 (7)	0.01016 (3)	0.0276 (2)	
O1	0.8581 (3)	1.1345 (5)	0.3581 (2)	0.0284 (9)	
N1	0.6444 (3)	0.7095 (5)	0.2424 (2)	0.0102 (8)	
N2	0.5954 (3)	0.4391 (5)	0.1799 (2)	0.0102 (8)	
N3	0.6749 (3)	0.9809 (5)	0.3027 (2)	0.0112 (8)	
H3A	0.598 (2)	0.977 (7)	0.310 (3)	0.019 (14)*	
C1	0.6261 (4)	0.3046 (6)	0.1387 (3)	0.0109 (9)	
C2	0.7405 (4)	0.2935 (6)	0.1146 (3)	0.0135 (9)	
H2	0.7594	0.1920	0.0870	0.016*	
C3	0.8233 (4)	0.4320 (6)	0.1320 (3)	0.0139 (10)	
H3	0.8999	0.4294	0.1152	0.017*	
C4	0.7944 (4)	0.5784 (6)	0.1749 (3)	0.0106 (9)	
C5	0.8705 (4)	0.7317 (6)	0.1931 (3)	0.0134 (9)	
H5	0.9469	0.7402	0.1763	0.016*	
C6	0.8334 (4)	0.8676 (6)	0.2351 (3)	0.0133 (10)	
H6	0.8830	0.9719	0.2474	0.016*	
C7	0.7196 (4)	0.8491 (6)	0.2596 (3)	0.0102 (9)	
C8	0.6797 (4)	0.5770 (6)	0.1993 (3)	0.0083 (9)	
C9	0.5270 (4)	0.1624 (6)	0.1145 (3)	0.0150 (10)	
H9A	0.4841	0.1468	0.1595	0.018*	
H9B	0.5680	0.0501	0.1069	0.018*	
C10	0.7442 (4)	1.1158 (6)	0.3487 (3)	0.0143 (10)	
C11	0.6664 (5)	1.2360 (6)	0.3869 (3)	0.0176 (10)	
H11A	0.7232	1.3202	0.4242	0.026*	
H11B	0.6211	1.1668	0.4195	0.026*	
H11C	0.6041	1.2992	0.3420	0.026*	
O1A	0.8985 (3)	0.9736 (4)	0.0366 (2)	0.0184 (7)	
C1A	1.0174 (4)	1.0481 (6)	0.0834 (3)	0.0166 (10)	
H1A1	1.0749	0.9539	0.1135	0.020*	
H1A2	1.0025	1.1303	0.1260	0.020*	
C2A	0.9197 (5)	0.8555 (7)	−0.0259 (3)	0.0206 (11)	
H2A1	0.8369	0.8059	−0.0594	0.025*	
H2A2	0.9751	0.7574	0.0020	0.025*	

### N-(7-Bromomethyl-1,8-naphthyridin-2-yl)acetamide dioxane hemisolvate (I) Atomic displacement parameters (Å^2^)

**Table:**

	*U*^11^	*U*^22^	*U*^33^	*U*^12^	*U*^13^	*U*^23^
Br1	0.0237 (3)	0.0352 (4)	0.0229 (3)	−0.0019 (2)	0.0047 (2)	−0.0049 (2)
O1	0.0186 (19)	0.033 (2)	0.037 (2)	−0.0123 (16)	0.0134 (17)	−0.0229 (18)
N1	0.0104 (18)	0.0098 (18)	0.0115 (19)	0.0001 (14)	0.0051 (15)	0.0003 (15)
N2	0.0113 (18)	0.0105 (19)	0.0100 (19)	−0.0009 (15)	0.0051 (15)	0.0007 (15)
N3	0.0088 (18)	0.0130 (19)	0.0137 (19)	−0.0012 (15)	0.0064 (15)	−0.0031 (15)
C1	0.013 (2)	0.012 (2)	0.007 (2)	0.0023 (18)	0.0016 (17)	0.0027 (17)
C2	0.016 (2)	0.015 (2)	0.010 (2)	0.0055 (18)	0.0037 (18)	−0.0025 (18)
C3	0.012 (2)	0.021 (3)	0.010 (2)	0.0032 (19)	0.0047 (18)	0.0000 (19)
C4	0.011 (2)	0.015 (2)	0.006 (2)	0.0039 (17)	0.0027 (17)	0.0032 (17)
C5	0.009 (2)	0.019 (2)	0.013 (2)	0.0012 (18)	0.0040 (18)	0.0026 (18)
C6	0.010 (2)	0.017 (2)	0.013 (2)	−0.0019 (18)	0.0033 (18)	0.0009 (18)
C7	0.011 (2)	0.012 (2)	0.007 (2)	0.0015 (17)	0.0012 (17)	0.0021 (17)
C8	0.010 (2)	0.013 (2)	0.002 (2)	0.0011 (17)	0.0022 (16)	0.0006 (17)
C9	0.019 (2)	0.015 (2)	0.012 (2)	0.0000 (19)	0.0050 (19)	−0.0037 (18)
C10	0.015 (2)	0.017 (2)	0.013 (2)	−0.0035 (18)	0.0070 (19)	−0.0039 (18)
C11	0.020 (3)	0.017 (3)	0.017 (2)	−0.0029 (19)	0.007 (2)	−0.006 (2)
O1A	0.0185 (17)	0.0224 (18)	0.0162 (17)	0.0009 (14)	0.0082 (14)	−0.0020 (14)
C1A	0.020 (2)	0.017 (2)	0.013 (2)	0.0031 (19)	0.004 (2)	−0.0062 (19)
C2A	0.021 (3)	0.021 (3)	0.020 (3)	−0.005 (2)	0.007 (2)	−0.002 (2)

### N-(7-Bromomethyl-1,8-naphthyridin-2-yl)acetamide dioxane hemisolvate (I) Geometric parameters (Å, º)

**Table:**

Br1—C9	1.938 (5)	C5—C6	1.370 (6)
O1—C10	1.214 (5)	C6—H6	0.9500
N1—C7	1.324 (6)	C6—C7	1.414 (6)
N1—C8	1.352 (5)	C9—H9A	0.9900
N2—C1	1.325 (6)	C9—H9B	0.9900
N2—C8	1.373 (6)	C10—C11	1.503 (6)
N3—C10	1.372 (6)	C11—H11A	0.9800
N3—C7	1.398 (6)	C11—H11B	0.9800
N3—H3A	0.883 (10)	C11—H11C	0.9800
C1—C9	1.502 (6)	O1A—C1A	1.426 (6)
C1—C2	1.412 (6)	O1A—C2A	1.438 (6)
C2—H2	0.9500	C1A—C2A^i^	1.510 (7)
C2—C3	1.366 (6)	C1A—H1A1	0.9900
C3—H3	0.9500	C1A—H1A2	0.9900
C3—C4	1.405 (6)	C2A—C1A^i^	1.510 (7)
C4—C8	1.416 (6)	C2A—H2A1	0.9900
C4—C5	1.415 (6)	C2A—H2A2	0.9900
C5—H5	0.9500		
			
C7—N1—C8	117.6 (4)	N2—C1—C9	115.4 (4)
C1—N2—C8	117.7 (4)	N1—C8—N2	115.1 (4)
C10—N3—C7	127.3 (4)	N1—C8—C4	123.2 (4)
C10—N3—H3A	110 (3)	N2—C8—C4	121.6 (4)
C7—N3—H3A	122 (3)	Br1—C9—H9A	110.0
N1—C7—N3	113.9 (4)	Br1—C9—H9B	110.0
N1—C7—C6	123.9 (4)	O1—C10—N3	122.8 (4)
N3—C7—C6	122.2 (4)	O1—C10—C11	123.4 (4)
C1—C2—H2	120.8	N3—C10—C11	113.7 (4)
C1—C9—H9A	110.0	C10—C11—H11A	109.5
C1—C9—H9B	110.0	C10—C11—H11B	109.5
C1—C9—Br1	108.4 (3)	H11A—C11—H11B	109.5
C2—C1—C9	120.4 (4)	C10—C11—H11C	109.5
C2—C3—C4	119.5 (4)	H11A—C11—H11C	109.5
C2—C3—H3	120.2	H11B—C11—H11C	109.5
C3—C2—C1	118.4 (4)	C1A—O1A—C2A	109.6 (3)
C3—C2—H2	120.8	O1A—C1A—C2A^i^	110.8 (4)
C3—C4—C5	124.3 (4)	O1A—C1A—H1A1	109.5
C3—C4—C8	118.5 (4)	C2A^i^—C1A—H1A1	109.5
C4—C3—H3	120.2	O1A—C1A—H1A2	109.5
C4—C5—H5	120.2	C2A^i^—C1A—H1A2	109.5
C5—C4—C8	117.1 (4)	H1A1—C1A—H1A2	108.1
C5—C6—C7	118.4 (4)	O1A—C2A—C1A^i^	109.9 (4)
C5—C6—H6	120.8	O1A—C2A—H2A1	109.7
C6—C5—C4	119.7 (4)	C1A^i^—C2A—H2A1	109.7
C6—C5—H5	120.2	O1A—C2A—H2A2	109.7
C7—C6—H6	120.8	C1A^i^—C2A—H2A2	109.7
H9A—C9—H9B	108.4	H2A1—C2A—H2A2	108.2
N2—C1—C2	124.1 (4)		
			
C8—N1—C7—N3	178.8 (4)	C5—C4—C8—N1	−3.6 (6)
C8—N1—C7—C6	0.4 (6)	C5—C4—C8—N2	175.9 (4)
C10—N3—C7—N1	160.8 (4)	C6—C5—C4—C3	179.7 (4)
C10—N3—C7—C6	−20.8 (7)	C6—C5—C4—C8	2.0 (6)
N1—C7—C6—C5	−1.9 (7)	C7—C6—C5—C4	0.5 (6)
N3—C7—C6—C5	179.9 (4)	C7—N1—C8—N2	−177.2 (4)
C1—N2—C8—C4	1.3 (6)	C7—N1—C8—C4	2.4 (6)
C1—N2—C8—N1	−179.1 (4)	C7—N3—C10—O1	−1.0 (8)
C2—C1—C9—Br1	−93.8 (4)	C7—N3—C10—C11	179.7 (4)
C3—C2—C1—N2	−2.4 (7)	C8—C4—C3—C2	0.3 (6)
C3—C2—C1—C9	174.8 (4)	C8—N2—C1—C2	0.9 (6)
C3—C4—C8—N1	178.5 (4)	C8—N2—C1—C9	−176.4 (4)
C3—C4—C8—N2	−1.9 (6)	N2—C1—C9—Br1	83.6 (4)
C4—C3—C2—C1	1.7 (7)	C2A—O1A—C1A—C2A^i^	58.6 (5)
C5—C4—C3—C2	−177.3 (4)	C1A—O1A—C2A—C1A^i^	−58.0 (5)

Symmetry code: (i) −*x*+2, −*y*+2, −*z*.

### N-(7-Bromomethyl-1,8-naphthyridin-2-yl)acetamide dioxane hemisolvate (I) Hydrogen-bond geometry (Å, º)

Cg1 and Cg2 are the centroids of rings N1/C1-C4/C8, and N2/C4-C8, respectively.

**Table:**

*D*—H···*A*	*D*—H	H···*A*	*D*···*A*	*D*—H···*A*
C6—H6···O1	0.95	2.29	2.836 (6)	116
N3—H3*A*···N2^ii^	0.88 (1)	2.18 (1)	3.053 (5)	173 (5)
C1*A*—H1*A*1···O1^iii^	0.99	2.55	3.462 (6)	153
C2—H2···O1*A*^iv^	0.95	2.54	3.438 (5)	157
C5—H5···O1^iii^	0.95	2.48	3.376 (6)	157
C9—H9*A*···N1^v^	0.99	2.47	3.418 (6)	161
C9—Br1···*Cg*1^vi^	1.94 (1)	3.70 (1)	5.563 (5)	161 (1)
C9—Br1···*Cg*2^vii^	1.94 (1)	3.70 (1)	5.436 (5)	148 (1)

Symmetry codes: (ii) −*x*+1, *y*+1/2, −*z*+1/2; (iii) −*x*+2, *y*−1/2, −*z*+1/2; (iv) *x*, *y*−1, *z*; (v) −*x*+1, *y*−1/2, −*z*+1/2; (vi) −*x*+1, −*y*+1, −*z*; (vii) −*x*+1, −*y*, −*z*.

### Bis[N-(7-dibromomethyl-1,8-naphthyridin-2-yl)acetamide] dioxane hemisolvate (II) Crystal data

**Table:**

2C_11_H_9_Br_2_N_3_O·0.5C_4_H_8_O_2_	*Z* = 2
*M**_r_* = 762.11	*F*(000) = 744
Triclinic, *P*1	*D*_x_ = 1.899 Mg m^−^^3^
*a* = 9.4065 (5) Å	Mo *K*α radiation, λ = 0.71073 Å
*b* = 9.5271 (5) Å	Cell parameters from 9921 reflections
*c* = 16.6464 (10) Å	θ = 2.4–29.1°
α = 88.777 (3)°	µ = 6.08 mm^−^^1^
β = 81.057 (2)°	*T* = 100 K
γ = 64.928 (2)°	Plate, colourless
*V* = 1333.15 (13) Å^3^	0.40 × 0.18 × 0.09 mm

### Bis[N-(7-dibromomethyl-1,8-naphthyridin-2-yl)acetamide] dioxane hemisolvate (II) Data collection

**Table:**

CCD area detector diffractometer	5247 reflections with *I* > 2σ(*I*)
φ and ω scans	*R*_int_ = 0.025
Absorption correction: multi-scan (SADABS; Bruker, 2014)	θ_max_ = 26.9°, θ_min_ = 2.4°
*T*_min_ = 0.195, *T*_max_ = 0.611	*h* = −11→11
32569 measured reflections	*k* = −12→12
5659 independent reflections	*l* = −21→21

### Bis[N-(7-dibromomethyl-1,8-naphthyridin-2-yl)acetamide] dioxane hemisolvate (II) Refinement

**Table:**

Refinement on *F*^2^	Primary atom site location: structure-invariant direct methods
Least-squares matrix: full	Secondary atom site location: difference Fourier map
*R*[*F*^2^ > 2σ(*F*^2^)] = 0.029	Hydrogen site location: mixed
*wR*(*F*^2^) = 0.074	H atoms treated by a mixture of independent and constrained refinement
*S* = 1.12	*w* = 1/[σ^2^(*F*_o_^2^) + (0.0205*P*)^2^ + 4.6947*P*] where *P* = (*F*_o_^2^ + 2*F*_c_^2^)/3
5659 reflections	(Δ/σ)_max_ = 0.001
354 parameters	Δρ_max_ = 0.92 e Å^−^^3^
6 restraints	Δρ_min_ = −0.80 e Å^−^^3^

### Bis[N-(7-dibromomethyl-1,8-naphthyridin-2-yl)acetamide] dioxane hemisolvate (II) Special details

**Table:**

Geometry. All esds (except the esd in the dihedral angle between two l.s. planes) are estimated using the full covariance matrix. The cell esds are taken into account individually in the estimation of esds in distances, angles and torsion angles; correlations between esds in cell parameters are only used when they are defined by crystal symmetry. An approximate (isotropic) treatment of cell esds is used for estimating esds involving l.s. planes.

### Bis[N-(7-dibromomethyl-1,8-naphthyridin-2-yl)acetamide] dioxane hemisolvate (II) Fractional atomic coordinates and isotropic or equivalent isotropic displacement parameters (Å^2^)

**Table:**

	*x*	*y*	*z*	*U*_iso_*/*U*_eq_	Occ. (<1)
Br1	0.65226 (4)	−0.27545 (4)	0.46303 (2)	0.02418 (9)	
Br2	0.50091 (4)	−0.43628 (4)	0.36111 (2)	0.01949 (8)	
O1	−0.1936 (3)	0.5684 (2)	0.70075 (14)	0.0193 (5)	
N1	0.2448 (3)	−0.0854 (3)	0.52469 (15)	0.0117 (5)	
N2	0.0579 (3)	0.1182 (3)	0.60845 (15)	0.0122 (5)	
N3	−0.1214 (3)	0.3074 (3)	0.70039 (16)	0.0136 (5)	
H3	−0.125 (5)	0.230 (3)	0.730 (2)	0.032 (12)*	
C1	0.3284 (3)	−0.1392 (3)	0.45189 (18)	0.0125 (6)	
C2	0.3087 (4)	−0.0548 (4)	0.38070 (18)	0.0149 (6)	
H2	0.3725	−0.1004	0.3299	0.018*	
C3	0.1948 (4)	0.0948 (4)	0.38720 (19)	0.0155 (6)	
H3AA	0.1775	0.1550	0.3404	0.019*	
C4	0.1032 (4)	0.1593 (3)	0.46373 (18)	0.0133 (6)	
C5	−0.0132 (4)	0.3143 (3)	0.4786 (2)	0.0157 (6)	
H5	−0.0388	0.3810	0.4346	0.019*	
C6	−0.0883 (4)	0.3676 (3)	0.55553 (19)	0.0142 (6)	
H6	−0.1660	0.4717	0.5662	0.017*	
C7	−0.0479 (3)	0.2642 (3)	0.61988 (18)	0.0127 (6)	
C8	0.1333 (3)	0.0649 (3)	0.53149 (18)	0.0113 (5)	
C9	0.4604 (4)	−0.2989 (3)	0.45387 (18)	0.0139 (6)	
H9	0.4325	−0.3480	0.5039	0.017*	
C10	−0.1954 (4)	0.4554 (3)	0.73566 (19)	0.0146 (6)	
C11	−0.2752 (4)	0.4634 (4)	0.8217 (2)	0.0192 (6)	
H11A	−0.1986	0.4466	0.8588	0.029*	
H11B	−0.3147	0.3831	0.8283	0.029*	
H11C	−0.3644	0.5658	0.8345	0.029*	
Br1A	0.80621 (4)	−0.30529 (4)	0.21420 (2)	0.02498 (9)	
Br2A	0.76639 (5)	−0.42214 (4)	0.04563 (2)	0.03087 (10)	
O1A	−0.3475 (3)	0.3086 (3)	0.21196 (14)	0.0213 (5)	
N1A	0.3820 (3)	−0.1632 (3)	0.17991 (15)	0.0114 (5)	
N2A	0.1123 (3)	−0.0240 (3)	0.21618 (14)	0.0108 (5)	
N3A	−0.1546 (3)	0.1001 (3)	0.26087 (16)	0.0131 (5)	
H3A	−0.139 (4)	0.027 (3)	0.2972 (17)	0.018 (9)*	
C1A	0.5218 (3)	−0.1707 (3)	0.14603 (17)	0.0121 (6)	
C2A	0.5460 (4)	−0.0467 (4)	0.10905 (19)	0.0168 (6)	
H2A	0.6490	−0.0594	0.0843	0.020*	
C3A	0.4166 (4)	0.0928 (4)	0.10975 (19)	0.0163 (6)	
H3AB	0.4288	0.1791	0.0856	0.020*	
C4A	0.2652 (4)	0.1076 (3)	0.14659 (18)	0.0123 (6)	
C5A	0.1237 (4)	0.2459 (3)	0.15230 (19)	0.0152 (6)	
H5A	0.1267	0.3378	0.1306	0.018*	
C6A	−0.0172 (4)	0.2474 (3)	0.18892 (19)	0.0142 (6)	
H6A	−0.1130	0.3399	0.1933	0.017*	
C7A	−0.0175 (3)	0.1079 (3)	0.22022 (17)	0.0121 (6)	
C8A	0.2525 (3)	−0.0247 (3)	0.18011 (17)	0.0100 (5)	
C9A	0.6575 (4)	−0.3261 (3)	0.15233 (18)	0.0149 (6)	
H9A	0.6132	−0.3952	0.1817	0.018*	
C10A	−0.3099 (4)	0.1975 (3)	0.25486 (18)	0.0137 (6)	
C11A	−0.4289 (4)	0.1510 (4)	0.30498 (19)	0.0166 (6)	
H11D	−0.5366	0.2314	0.3038	0.025*	
H11E	−0.4088	0.1389	0.3613	0.025*	
H11F	−0.4190	0.0524	0.2825	0.025*	
O1B	0.1348 (3)	0.0280 (3)	1.00203 (17)	0.0237 (7)	0.890 (5)
C1B	0.0015 (15)	0.1333 (9)	0.9682 (7)	0.0242 (16)	0.890 (5)
H1B1	0.0192	0.1083	0.9090	0.029*	0.890 (5)
H1B2	−0.0083	0.2401	0.9755	0.029*	0.890 (5)
C2B	0.150 (2)	−0.1267 (10)	0.9930 (6)	0.0223 (10)	0.890 (5)
H2B1	0.2389	−0.1990	1.0191	0.027*	0.890 (5)
H2B2	0.1721	−0.1587	0.9345	0.027*	0.890 (5)
O1BA	0.099 (3)	−0.003 (3)	0.9249 (13)	0.0237 (7)	0.110 (5)
C1BA	−0.008 (13)	0.147 (8)	0.960 (7)	0.0242 (16)	0.110 (5)
H1B3	−0.0491	0.2215	0.9175	0.029*	0.110 (5)
H1B4	0.0424	0.1879	0.9953	0.029*	0.110 (5)
C2BA	0.146 (18)	−0.107 (10)	0.988 (5)	0.0223 (10)	0.110 (5)
H2B3	0.1887	−0.0616	1.0262	0.027*	0.110 (5)
H2B4	0.2343	−0.2048	0.9640	0.027*	0.110 (5)

### Bis[N-(7-dibromomethyl-1,8-naphthyridin-2-yl)acetamide] dioxane hemisolvate (II) Atomic displacement parameters (Å^2^)

**Table:**

	*U*^11^	*U*^22^	*U*^33^	*U*^12^	*U*^13^	*U*^23^
Br1	0.01616 (16)	0.02553 (18)	0.03175 (19)	−0.00758 (13)	−0.01019 (13)	0.00084 (14)
Br2	0.02109 (16)	0.01804 (16)	0.01863 (16)	−0.00768 (12)	−0.00289 (12)	−0.00092 (12)
O1	0.0206 (12)	0.0118 (10)	0.0266 (12)	−0.0073 (9)	−0.0054 (9)	0.0013 (9)
N1	0.0120 (12)	0.0130 (12)	0.0119 (12)	−0.0066 (10)	−0.0036 (9)	0.0025 (9)
N2	0.0119 (12)	0.0109 (12)	0.0149 (12)	−0.0055 (10)	−0.0034 (10)	0.0026 (9)
N3	0.0124 (12)	0.0104 (12)	0.0162 (13)	−0.0033 (10)	−0.0022 (10)	0.0022 (10)
C1	0.0118 (14)	0.0133 (14)	0.0137 (14)	−0.0062 (11)	−0.0040 (11)	0.0020 (11)
C2	0.0164 (15)	0.0185 (15)	0.0107 (14)	−0.0078 (12)	−0.0037 (11)	0.0013 (11)
C3	0.0195 (15)	0.0183 (15)	0.0139 (14)	−0.0111 (13)	−0.0083 (12)	0.0051 (12)
C4	0.0149 (14)	0.0133 (14)	0.0157 (15)	−0.0088 (12)	−0.0059 (11)	0.0026 (11)
C5	0.0167 (15)	0.0129 (14)	0.0205 (16)	−0.0079 (12)	−0.0073 (12)	0.0054 (12)
C6	0.0137 (14)	0.0102 (13)	0.0195 (15)	−0.0050 (11)	−0.0059 (12)	0.0035 (11)
C7	0.0115 (14)	0.0119 (14)	0.0168 (15)	−0.0069 (11)	−0.0026 (11)	0.0019 (11)
C8	0.0109 (13)	0.0115 (13)	0.0138 (14)	−0.0065 (11)	−0.0038 (11)	0.0028 (11)
C9	0.0139 (14)	0.0137 (14)	0.0127 (14)	−0.0043 (12)	−0.0030 (11)	0.0003 (11)
C10	0.0102 (14)	0.0121 (14)	0.0207 (16)	−0.0032 (11)	−0.0049 (11)	−0.0011 (12)
C11	0.0179 (16)	0.0149 (15)	0.0231 (17)	−0.0059 (12)	−0.0014 (13)	−0.0025 (12)
Br1A	0.01866 (17)	0.02336 (17)	0.03252 (19)	−0.00674 (13)	−0.01008 (14)	0.00544 (14)
Br2A	0.0356 (2)	0.02085 (17)	0.01875 (17)	0.00176 (15)	0.00427 (14)	−0.00107 (13)
O1A	0.0134 (11)	0.0211 (12)	0.0242 (12)	−0.0023 (9)	−0.0046 (9)	0.0091 (10)
N1A	0.0120 (12)	0.0110 (11)	0.0096 (11)	−0.0032 (10)	−0.0016 (9)	0.0005 (9)
N2A	0.0104 (12)	0.0127 (12)	0.0089 (11)	−0.0043 (10)	−0.0022 (9)	0.0010 (9)
N3A	0.0105 (12)	0.0112 (12)	0.0149 (13)	−0.0019 (10)	−0.0028 (10)	0.0043 (9)
C1A	0.0115 (14)	0.0122 (14)	0.0101 (13)	−0.0024 (11)	−0.0022 (11)	−0.0005 (10)
C2A	0.0136 (15)	0.0181 (15)	0.0175 (15)	−0.0068 (12)	0.0010 (12)	0.0014 (12)
C3A	0.0182 (15)	0.0141 (14)	0.0176 (15)	−0.0082 (12)	−0.0021 (12)	0.0032 (12)
C4A	0.0142 (14)	0.0123 (14)	0.0109 (13)	−0.0055 (11)	−0.0035 (11)	0.0000 (11)
C5A	0.0177 (15)	0.0122 (14)	0.0162 (15)	−0.0064 (12)	−0.0041 (12)	0.0034 (11)
C6A	0.0142 (14)	0.0087 (13)	0.0178 (15)	−0.0026 (11)	−0.0044 (12)	−0.0003 (11)
C7A	0.0126 (14)	0.0125 (14)	0.0104 (13)	−0.0041 (11)	−0.0036 (11)	0.0002 (11)
C8A	0.0111 (13)	0.0102 (13)	0.0079 (13)	−0.0033 (11)	−0.0027 (10)	−0.0011 (10)
C9A	0.0124 (14)	0.0149 (14)	0.0142 (14)	−0.0036 (12)	0.0006 (11)	−0.0001 (11)
C10A	0.0114 (14)	0.0149 (14)	0.0112 (14)	−0.0019 (11)	−0.0019 (11)	−0.0023 (11)
C11A	0.0116 (14)	0.0181 (15)	0.0182 (15)	−0.0048 (12)	−0.0018 (12)	0.0008 (12)
O1B	0.0220 (14)	0.0322 (15)	0.0239 (15)	−0.0181 (12)	−0.0031 (11)	−0.0030 (11)
C1B	0.034 (3)	0.025 (2)	0.019 (4)	−0.0170 (18)	−0.007 (2)	0.004 (2)
C2B	0.0226 (19)	0.023 (4)	0.021 (2)	−0.008 (3)	−0.0051 (17)	−0.0025 (18)
O1BA	0.0220 (14)	0.0322 (15)	0.0239 (15)	−0.0181 (12)	−0.0031 (11)	−0.0030 (11)
C1BA	0.034 (3)	0.025 (2)	0.019 (4)	−0.0170 (18)	−0.007 (2)	0.004 (2)
C2BA	0.0226 (19)	0.023 (4)	0.021 (2)	−0.008 (3)	−0.0051 (17)	−0.0025 (18)

### Bis[N-(7-dibromomethyl-1,8-naphthyridin-2-yl)acetamide] dioxane hemisolvate (II) Geometric parameters (Å, º)

**Table:**

Br1—C9	1.939 (3)	N3A—C7A	1.391 (4)
Br2—C9	1.929 (3)	N3A—H3A	0.892 (10)
O1—C10	1.218 (4)	C1A—C2A	1.407 (4)
N1—C1	1.318 (4)	C1A—C9A	1.505 (4)
N1—C8	1.364 (4)	C2A—C3A	1.368 (4)
N2—C7	1.320 (4)	C2A—H2A	0.9500
N2—C8	1.357 (4)	C3A—C4A	1.410 (4)
N3—C10	1.376 (4)	C3A—H3AB	0.9500
N3—C7	1.392 (4)	C4A—C8A	1.411 (4)
N3—H3	0.890 (10)	C4A—C5A	1.413 (4)
C1—C2	1.410 (4)	C5A—C6A	1.364 (4)
C1—C9	1.506 (4)	C5A—H5A	0.9500
C2—C3	1.366 (4)	C6A—C7A	1.418 (4)
C2—H2	0.9500	C6A—H6A	0.9500
C3—C4	1.409 (4)	C9A—H9A	1.0000
C3—H3AA	0.9500	C10A—C11A	1.504 (4)
C4—C5	1.415 (4)	C11A—H11D	0.9800
C4—C8	1.415 (4)	C11A—H11E	0.9800
C5—C6	1.357 (5)	C11A—H11F	0.9800
C5—H5	0.9500	O1B—C1B	1.423 (8)
C6—C7	1.423 (4)	O1B—C2B	1.430 (8)
C6—H6	0.9500	C1B—C2B^i^	1.49 (2)
C9—H9	1.0000	C1B—H1B1	0.9900
C10—C11	1.498 (5)	C1B—H1B2	0.9900
C11—H11A	0.9800	C2B—C1B^i^	1.49 (2)
C11—H11B	0.9800	C2B—H2B1	0.9900
C11—H11C	0.9800	C2B—H2B2	0.9900
Br1A—C9A	1.936 (3)	O1BA—C1BA	1.419 (10)
Br2A—C9A	1.937 (3)	O1BA—C2BA	1.420 (10)
O1A—C10A	1.218 (4)	C1BA—C2BA^i^	1.6 (2)
N1A—C1A	1.321 (4)	C1BA—H1B3	0.9900
N1A—C8A	1.364 (4)	C1BA—H1B4	0.9900
N2A—C7A	1.324 (4)	C2BA—C1BA^i^	1.6 (2)
N2A—C8A	1.359 (4)	C2BA—H2B3	0.9900
N3A—C10A	1.377 (4)	C2BA—H2B4	0.9900
			
C1—N1—C8	117.0 (3)	C4A—C3A—H3AB	120.2
C7—N2—C8	118.1 (3)	C3A—C4A—C8A	118.1 (3)
C10—N3—C7	126.9 (3)	C3A—C4A—C5A	124.7 (3)
C10—N3—H3	118 (3)	C8A—C4A—C5A	117.1 (3)
C7—N3—H3	115 (3)	C6A—C5A—C4A	120.0 (3)
N1—C1—C2	125.1 (3)	C6A—C5A—H5A	120.0
N1—C1—C9	112.1 (3)	C4A—C5A—H5A	120.0
C2—C1—C9	122.5 (3)	C5A—C6A—C7A	118.5 (3)
C3—C2—C1	117.9 (3)	C5A—C6A—H6A	120.7
C3—C2—H2	121.1	C7A—C6A—H6A	120.7
C1—C2—H2	121.1	N2A—C7A—N3A	114.2 (3)
C2—C3—C4	119.6 (3)	N2A—C7A—C6A	123.4 (3)
C2—C3—H3AA	120.2	N3A—C7A—C6A	122.4 (3)
C4—C3—H3AA	120.2	N2A—C8A—N1A	114.9 (3)
C3—C4—C5	124.7 (3)	N2A—C8A—C4A	123.1 (3)
C3—C4—C8	118.0 (3)	N1A—C8A—C4A	122.0 (3)
C5—C4—C8	117.2 (3)	C1A—C9A—Br1A	110.6 (2)
C6—C5—C4	120.0 (3)	C1A—C9A—Br2A	111.2 (2)
C6—C5—H5	120.0	Br1A—C9A—Br2A	110.01 (15)
C4—C5—H5	120.0	C1A—C9A—H9A	108.3
C5—C6—C7	118.5 (3)	Br1A—C9A—H9A	108.3
C5—C6—H6	120.7	Br2A—C9A—H9A	108.3
C7—C6—H6	120.7	O1A—C10A—N3A	123.5 (3)
N2—C7—N3	114.0 (3)	O1A—C10A—C11A	123.1 (3)
N2—C7—C6	123.3 (3)	N3A—C10A—C11A	113.4 (3)
N3—C7—C6	122.6 (3)	C10A—C11A—H11D	109.5
N2—C8—N1	114.9 (3)	C10A—C11A—H11E	109.5
N2—C8—C4	122.7 (3)	H11D—C11A—H11E	109.5
N1—C8—C4	122.3 (3)	C10A—C11A—H11F	109.5
C1—C9—Br2	115.1 (2)	H11D—C11A—H11F	109.5
C1—C9—Br1	107.8 (2)	H11E—C11A—H11F	109.5
Br2—C9—Br1	109.38 (15)	C1B—O1B—C2B	110.0 (8)
C1—C9—H9	108.1	O1B—C1B—C2B^i^	112.0 (10)
Br2—C9—H9	108.1	O1B—C1B—H1B1	109.2
Br1—C9—H9	108.1	C2B^i^—C1B—H1B1	109.2
O1—C10—N3	123.2 (3)	O1B—C1B—H1B2	109.2
O1—C10—C11	123.3 (3)	C2B^i^—C1B—H1B2	109.2
N3—C10—C11	113.5 (3)	H1B1—C1B—H1B2	107.9
C10—C11—H11A	109.5	O1B—C2B—C1B^i^	109.4 (10)
C10—C11—H11B	109.5	O1B—C2B—H2B1	109.8
H11A—C11—H11B	109.5	C1B^i^—C2B—H2B1	109.8
C10—C11—H11C	109.5	O1B—C2B—H2B2	109.8
H11A—C11—H11C	109.5	C1B^i^—C2B—H2B2	109.8
H11B—C11—H11C	109.5	H2B1—C2B—H2B2	108.2
C1A—N1A—C8A	117.6 (3)	C1BA—O1BA—C2BA	109 (8)
C7A—N2A—C8A	117.9 (3)	O1BA—C1BA—C2BA^i^	100 (9)
C10A—N3A—C7A	127.5 (3)	O1BA—C1BA—H1B3	111.8
C10A—N3A—H3A	117 (2)	C2BA^i^—C1BA—H1B3	111.8
C7A—N3A—H3A	115 (2)	O1BA—C1BA—H1B4	111.8
N1A—C1A—C2A	124.6 (3)	C2BA^i^—C1BA—H1B4	111.8
N1A—C1A—C9A	113.4 (3)	H1B3—C1BA—H1B4	109.5
C2A—C1A—C9A	122.1 (3)	O1BA—C2BA—C1BA^i^	116 (9)
C3A—C2A—C1A	118.0 (3)	O1BA—C2BA—H2B3	108.4
C3A—C2A—H2A	121.0	C1BA^i^—C2BA—H2B3	108.4
C1A—C2A—H2A	121.0	O1BA—C2BA—H2B4	108.3
C2A—C3A—C4A	119.6 (3)	C1BA^i^—C2BA—H2B4	108.3
C2A—C3A—H3AB	120.2	H2B3—C2BA—H2B4	107.4
			
C8—N1—C1—C2	−1.6 (4)	N1A—C1A—C2A—C3A	1.6 (5)
C8—N1—C1—C9	173.1 (2)	C9A—C1A—C2A—C3A	−176.7 (3)
N1—C1—C2—C3	0.4 (5)	C1A—C2A—C3A—C4A	−0.3 (5)
C9—C1—C2—C3	−173.8 (3)	C2A—C3A—C4A—C8A	−1.4 (4)
C1—C2—C3—C4	0.5 (4)	C2A—C3A—C4A—C5A	178.9 (3)
C2—C3—C4—C5	177.3 (3)	C3A—C4A—C5A—C6A	179.6 (3)
C2—C3—C4—C8	−0.1 (4)	C8A—C4A—C5A—C6A	−0.1 (4)
C3—C4—C5—C6	−176.0 (3)	C4A—C5A—C6A—C7A	−0.3 (4)
C8—C4—C5—C6	1.4 (4)	C8A—N2A—C7A—N3A	−177.6 (2)
C4—C5—C6—C7	−0.5 (4)	C8A—N2A—C7A—C6A	0.0 (4)
C8—N2—C7—N3	178.7 (3)	C10A—N3A—C7A—N2A	−159.0 (3)
C8—N2—C7—C6	0.9 (4)	C10A—N3A—C7A—C6A	23.4 (5)
C10—N3—C7—N2	157.9 (3)	C5A—C6A—C7A—N2A	0.4 (5)
C10—N3—C7—C6	−24.3 (5)	C5A—C6A—C7A—N3A	177.8 (3)
C5—C6—C7—N2	−0.8 (5)	C7A—N2A—C8A—N1A	178.3 (2)
C5—C6—C7—N3	−178.3 (3)	C7A—N2A—C8A—C4A	−0.5 (4)
C7—N2—C8—N1	177.8 (3)	C1A—N1A—C8A—N2A	−179.7 (3)
C7—N2—C8—C4	0.1 (4)	C1A—N1A—C8A—C4A	−0.8 (4)
C1—N1—C8—N2	−175.7 (3)	C3A—C4A—C8A—N2A	−179.2 (3)
C1—N1—C8—C4	2.0 (4)	C5A—C4A—C8A—N2A	0.5 (4)
C3—C4—C8—N2	176.4 (3)	C3A—C4A—C8A—N1A	2.0 (4)
C5—C4—C8—N2	−1.2 (4)	C5A—C4A—C8A—N1A	−178.2 (3)
C3—C4—C8—N1	−1.2 (4)	N1A—C1A—C9A—Br1A	−118.3 (2)
C5—C4—C8—N1	−178.8 (3)	C2A—C1A—C9A—Br1A	60.3 (3)
N1—C1—C9—Br2	143.1 (2)	N1A—C1A—C9A—Br2A	119.2 (2)
C2—C1—C9—Br2	−42.1 (4)	C2A—C1A—C9A—Br2A	−62.3 (3)
N1—C1—C9—Br1	−94.6 (3)	C7A—N3A—C10A—O1A	−1.1 (5)
C2—C1—C9—Br1	80.3 (3)	C7A—N3A—C10A—C11A	177.5 (3)
C7—N3—C10—O1	−6.4 (5)	C2B—O1B—C1B—C2B^i^	58.1 (11)
C7—N3—C10—C11	174.9 (3)	C1B—O1B—C2B—C1B^i^	−56.6 (11)
C8A—N1A—C1A—C2A	−1.1 (4)	C2BA—O1BA—C1BA—C2BA^i^	−58 (10)
C8A—N1A—C1A—C9A	177.4 (2)	C1BA—O1BA—C2BA—C1BA^i^	68 (11)

Symmetry code: (i) −*x*, −*y*, −*z*+2.

### Bis[N-(7-dibromomethyl-1,8-naphthyridin-2-yl)acetamide] dioxane hemisolvate (II) Hydrogen-bond geometry (Å, º)

Cg1, Cg2 and Cg4 are the centroids of rings N1/C1-C4/C8, N2/C4-C8 and N2A/C4A-C8A, respectively.

**Table:**

*D*—H···*A*	*D*—H	H···*A*	*D*···*A*	*D*—H···*A*
C6—H6···O1	0.95	2.37	2.886 (4)	113
C6*A*—H6*A*···O1*A*	0.95	2.32	2.871 (5)	116
N3—H3···N2*A*^ii^	0.89 (3)	2.10 (3)	2.985 (4)	171 (4)
N3*A*—H3*A*···N2^ii^	0.89 (3)	2.09 (3)	2.950 (4)	163 (3)
C2—H2···N1*A*	0.95	2.56	3.397 (4)	147
C2*A*—H2*A*···O1*B*^iii^	0.95	2.42	3.344 (5)	163
C11—H11*B*···N1*A*^ii^	0.98	2.44	3.411 (5)	169
C11*A*—H11*D*···O1^iv^	0.98	2.46	3.437 (5)	179
C11*A*—H11*E*···N1^ii^	0.98	2.54	3.459 (4)	156
C3—H3*AA*···*Cg*4	0.95	2.82	3.548 (3)	134
C2*BA*—H2*B*3···*Cg*4^v^	0.99	2.96	3.82 (9)	145
C9—Br1···*Cg*1^iii^	1.94 (1)	3.62 (1)	5.270 (4)	141 (1)
C9—Br1···*Cg*2^iii^	1.94 (1)	3.32 (1)	5.247 (4)	173 (1)

Symmetry codes: (ii) −*x*, −*y*, −*z*+1; (iii) −*x*+1, −*y*, −*z*+1; (iv) −*x*−1, −*y*+1, −*z*+1; (v) *x*, *y*, *z*+1.
